# Surface Electromyographic Biofeedback and the Effortful Swallow Exercise for Stroke-Related Dysphagia and in Healthy Ageing

**DOI:** 10.1007/s00455-020-10129-8

**Published:** 2020-05-22

**Authors:** Sally K. Archer, Christina H. Smith, Di J. Newham

**Affiliations:** 1 Centre of Human and Aerospace Physiological Sciences, King’s College London, London, UK; 2 Speech and Language Therapy Department, Guy’s and St Thomas’ NHS Foundation Trust, Westminster Bridge Road, London, SE1 7EH UK; 3 NIHR Biomedical Research Centre at Guy’s and St Thomas’ NHS Foundation Trust and King’s College London, London, UK; 4 Division of Psychology and Language Sciences, University College London, London, UK

**Keywords:** Dysphagia, Deglutition, Deglutition disorders, Speech and language therapy, Biofeedback, Surface electromyography, Effortful swallow, Stroke

## Abstract

Dysphagia is common after stroke, leading to adverse outcome. The Effortful Swallow (ES) is recommended to improve swallowing but it is not known if dysphagic patients can increase muscle activity during the exercise or if age affects performance. Providing surface electromyographic (sEMG) biofeedback during dysphagia therapy may enhance exercise completion, but this has not been investigated and the technique’s acceptability to patients is not known. Aims: To determine if age or post-stroke dysphagia affect the ability to increase submental muscle activity during the ES, if sEMG biofeedback improves ES performance and if sEMG is an acceptable addition to therapy. In a Phase I study submental sEMG amplitudes were measured from 15 people with dysphagia < 3 months post-stroke and 85 healthy participants aged 18–89 years during swallowing (NS) and when they performed the ES with and without sEMG biofeedback. Participant feedback was collected via questionnaire. Measurements were compared with repeated measures ANOVA and age effects were examined with linear regression. Both groups produced significantly greater muscle activity for the ES than NS (*p* < 0.001) and significantly increased activity with biofeedback (*p* < 0.001) with no effect of age. Participant feedback about sEMG was very positive; over 98% would be happy to use it regularly. The ES is a physiologically beneficial dysphagia exercise, increasing muscle activity during swallowing. sEMG biofeedback further enhances performance and is considered an acceptable technique by patients. These findings support the potential application of sEMG biofeedback and the ES in dysphagia therapy in stroke, justifying further investigation of patient outcome.

## Introduction

Oropharyngeal dysphagia, or difficulty with swallowing, affects around 50% of acute stroke patients [1], is associated with an 11-fold increase in the risk of pneumonia [2], is an independent predictor of mortality and is associated with poor nutrition, dehydration, increased length of stay, institutionalisation and poor quality of life [3–6].

Speech and language therapists (SLTs) recommend behavioural therapy techniques for dysphagia in which patients work to gain volitional control of previously automatic movements with the aim of restoring swallowing ability, airway protection and quality of life [7]. Frequently, patients are asked to learn and practise movements that are novel and/or difficult to monitor as part of behavioural swallowing rehabilitation [8]. Feedback is vital for motor learning to be successful as the learner adapts subsequent behaviour according to the difference between the actual and the desired output [9–11]. It is accepted that individuals generate motor commands that will maximise the reward they receive [11], so it follows that accurate feedback is essential and the right behaviour is rewarded to shape learning. However, feedback is challenging to deliver in dysphagia therapy when there is no overt sign of successful accomplishment of a target. Clinical swallowing assessments have poor reliability [12] so it likely that feedback provided during therapy may lack validity. This has implications for ensuring that optimal movements are reinforced and for motivating the patient to continue trying.

A solution could be incorporating biofeedback into therapy programmes. This involves taking measurements of a chosen physical function and displaying them directly or through a feedback signal so that the patient can practise controlling the signal by altering their movement or behaviour. This enables small changes in physiological processes to be noticed and reinforced so that behaviour can be modified [13].

Surface electromyography (sEMG) provides a type of neuromuscular biofeedback by displaying a visual or auditory representation of muscle activity. Electrodes are placed on the skin and detect motor unit action potentials generated by muscle contraction. With increasing force of muscle contraction, there is successive activation of motor units and an increase in the firing rate of all motor units recruited [14] leading to an increase in the amplitude of the sEMG signal, which can be displayed graphically. By using this feedback, patients can work to increase muscle activity [15].

Biofeedback has been incorporated into stroke rehabilitation for decades [15] with good evidence to suggest it leads to improvements in limb function and gait following stroke [16–18]. It is thought to work best when used with functions that are not normally directly observable [19]. Therefore incorporating biofeedback in dysphagia rehabilitation would provide the patient with direct information on a complex and subtle process to improve motor control for swallowing, while potentially enabling more active involvement and thereby improving outcome [20].

Increased conscious control for swallowing with biofeedback was indicated by the results of an fMRI study in which visual feedback during swallowing led to increased activation in frontal regions of the brain, indicating that the feedback directed more attention to motor planning [21]. Several studies have reported benefits of swallowing therapy with adjunctive sEMG biofeedback in dysphagic stroke patients [8, 22–27]. However, many of these studies are retrospective and/or case studies and none used a control group, blinding or randomisation and the sample sizes are small. Furthermore, most do not follow a specified, structured treatment protocol, used mixed treatments [8, 22, 24] and include mixed populations [8, 20]. These methodological weaknesses limit the interpretation of the reported findings. Indeed, a recent robust systematic review and meta-analysis concluded that there was a paucity of good quality studies examining the effect of biofeedback in dysphagia therapy [28].

The “effortful swallow” (ES) is a commonly recommended exercise [29] in which the patient is typically instructed to swallow while pushing hard with the tongue on the palate and “squeezing hard” with their swallowing muscles [30]. It is a task-specific exercise that aims to increase posterior tongue base movement, drive the bolus more efficiently through the pharynx, reduce post-swallow residue and reduce the incidence of aspiration [31, 32]. Healthy participants produce significantly greater muscle activity during the ES than the normal swallow (NS) [30, 31, 33, 34]. It is not clear if this capacity to increase the activity of the swallowing muscles beyond the level required for regular swallowing diminishes with age. This is despite evidence of pharyngeal muscle atrophy and reduced functional reserve, i.e. the difference between the amount of muscle activity used for a particular task and the maximal effort that that can be obtained, in lingual and pharyngeal pressure generation with healthy ageing [35–37].

There is a loss of muscle strength (atrophy) following stroke which results partly from activation failure due to direct neurological effects and also loss of bulk [38, 39]. Therefore an impaired ability to maximally drive the swallowing muscles following stroke, together with intrinsic muscle weakness, may affect dysphagic patients’ ability to increase muscle activity for the ES. While the ES is routinely prescribed to dysphagic stroke patients [39], their ability to increase muscle activity and therefore modify sEMG amplitudes during the ES, has not been examined and therefore its physiological benefit is not known. It follows that should patients be unable to modify the sEMG trace for the ES, they may be unable to benefit from incorporating sEMG for biofeedback in therapy.

Using biofeedback in therapy relies on the patient taking an active role in order to alter their behaviour in response to the feedback. It is not known whether dysphagic patients can actually use and interpret biofeedback for swallowing to improve exercise performance, or whether they find it an acceptable part of therapy. There remains a need for studies that examine the physiological and functional benefits of biofeedback in swallowing therapy and also whether dysphagic patients can interpret swallowing biofeedback to improve functional performance. The National Institute for Health and Clinical Excellence guidelines on patient experience states that care should take into account patients’ feedback and views on treatments [40]. Therefore it is important to explore participants’ own experiences of sEMG biofeedback to enable comprehensive evaluation of its role in dysphagia therapy.

## Objectives

This preliminary (Phase I) study sought to determine:

1. If age or stroke-related dysphagia affect the ability to increase submental muscle activity during the ES relative to habitual/normal swallowing (NS).
2. If sEMG biofeedback improves the performance of the ES by healthy and dysphagic stroke participants.
3. If participants find sEMG comfortable and helpful and whether they consider it would be an acceptable part of regular therapy.

## Methods

Full ethical and R&D approvals were obtained for the study and informed written consent was gained from all participants.

## Study Design

A Phase I observational study was conducted.

## Participants

### Healthy Participants

Healthy volunteers were recruited from King’s College London, and Guy’s and St Thomas’ NHS Foundation Trust, UK. Inclusion criteria were age > 18 years and the ability to eat and drink a normal diet and fluids with no difficulty, determined by questioning and Functional Oral Intake Scale (FOIS), a validated 7-point scale that measures the functional severity of dysphagia [41]. Exclusion criteria were any history of dysphagia, stroke or other neurological or neuromuscular illness or head and neck cancer or surgery as determined by questioning. All healthy participants recruited were included in the study of the effects of ageing and this group was called “healthy participants”. The first healthy participants recruited who were aged > 65 years were also allocated to form a healthy age-matched control group for direct comparison to stroke participants; this group was called “healthy controls”.

### Stroke Participants

Fifteen consecutive dysphagic acute stroke participants were recruited from the Stroke Unit at Guy’s and St Thomas’ Hospital NHS Foundation Trust. All those referred to SLT for swallowing assessment were approached. Inclusion criteria were ≤ 3 months post first stroke, referral to SLT for assessment and management of dysphagia, presence of dysphagia on Fibreoptic Endoscopic Evaluation of Swallowing (FEES) incorporating the Rosenbek Penetration-Aspiration scale [42], FOIS < 6 (i.e. requiring modification and/or restriction of oral intake); and ability to give informed consent with supported/total communication if necessary (as determined by their medical consultant and SLT). Exclusion criteria were FOIS score ≥ 6, any previous history of dysphagia, stroke, neurological illness and/or head and neck cancer or surgery.

## Procedure

On recruitment to the study, the Barthel Index [43] was recorded for stroke participants and their swallowing was assessed with FEES following a standard protocol. Aspiration was assessed with the Penetration Aspiration Scale [42].

It was anticipated that a greater effect of biofeedback might be noted with practice and/or participants’ views on the technique would be better established with time and therefore it was important to incorporate more than one session in the study design. Data was therefore collected over two sessions for all participants in order to assess reproducibility and to better establish the feasibility of the technique. In order to control for a learning effect masking the impact of biofeedback, participants were randomised as to whether they completed the tasks in the “with biofeedback” or “without biofeedback” condition first.

### Electrode Placement

**Fig. 1:**
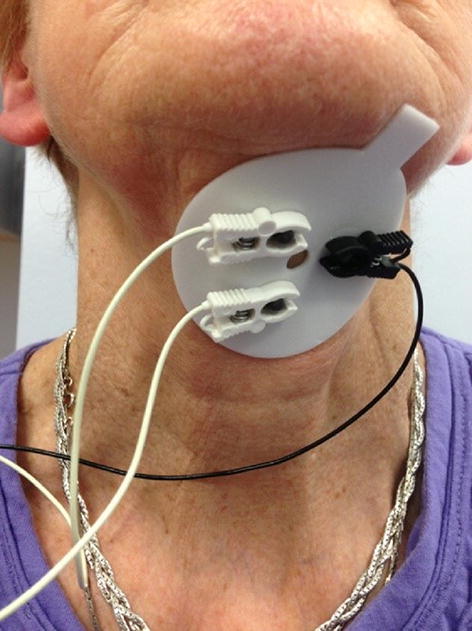
Electrode placement. The two recording electrodes were positioned longitudinally on the anterior neck, mid-way between the mental spine of the mandible and the hyoid bone (attached to white wires), with the reference electrode to the side (attached to black wire). The self-adhesive electrode discs were then taped in place (Micropore, MidMeds, Waltham Abbey, UK; not shown)

Prior to electrode placement, the skin was prepared by light abrasion and cleaning with chlorhexidine/alcohol wipes. EMG signals were recorded with the standard electrodes supplied by KayPentax for use with the Digital Swallow Workstation (DSW, KayPentax, NJ, USA); disposable circular adhesive electrode disks (57.2 mm in diameter) with three Ag/AgCl electrodes (diameter 12 mm). After application of electrode gel, two recording electrodes were placed longitudinally (inter-electrode distance 20 mm centre to centre) on the anterior neck, mid-way between the mental spine of the mandible and the hyoid bone, with the reference electrode to the side and taped in place (Fig. 1). This configuration detects collective activity from bilateral submental muscles (mylohyoid, geniohyoid and anterior belly of the digastrics) [30]. The disks were taped in place (Micropore, MidMeds, Waltham Abbey, UK). Measurements were taken to ensure consistent positioning of electrodes between sessions and individuals.

### sEMG Signal Processing

Signals were sampled at 1 kHz and automatically processed with the DSW in-built Swallow Signals Lab (SSL) and software i.e. filtered with a bandwidth of 50–220 Hz and a 12 dB/octave rolloff, full-wave rectified and then low passed filtered at 3 Hz.

### Swallow Tasks

All participants were taught the ES and were instructed to “swallow hard, squeezing all of your throat muscles and pushing hard with your tongue on the roof of your mouth” [30, 44]. For the NS, participants were asked to “swallow in your normal way”. For each task, participants were given 5 ml boluses of water from a teaspoon and asked to hold the water in their mouth until asked to swallow. Those considered at high risk of aspiration on water from the FEES assessment, were given a teaspoon of their safest consistency or moistened mouth care swabs if they were nil by mouth, and the same bolus type was used across sessions.

### Session Procedure

Prior to sEMG recording, the researcher (SA) observed participants practising the ES and palpated their laryngeal movement until she felt they had mastered it. Participants were randomised to complete the tasks with or without biofeedback first and repeated a series of swallow tasks in each condition in the following order: three normal swallows then 6 effortful swallows.

There was a 30 s rest between each bolus and the sequence was repeated after a 5 min rest so that each participant completed the series both with and without biofeedback. Biofeedback involved the participant watching the DSW screen while they completed the tasks. They were orientated to the information on the screen and verbally encouraged to increase the amplitude of the activity trace for each successive ES with cursors placed on the preceding attempt to give them a visual target to “beat”. For the NS, no additional instructions were given. In the condition without biofeedback, participants completed the tasks with the DSW screen turned away from them while general verbal encouragement was given to swallow “harder”. All participants were then invited to return for a second identical session in which the protocol was repeated. Sessions were scheduled > 24 h apart but within one week of each other to minimise the degree of change in swallowing status in stroke participants.

### Questionnaire

At the end of the second session, participants completed a questionnaire in which they were asked 8 questions about their impression of completing the ES with and without sEMG feedback (Table 1). Responses to questions 1 to 3 were made on a 5-point Likert-style scale from *very easy* to *very difficult* and for question 5 on a 4-point scale from *very comfortable* to *very uncomfortable.* The questionnaire was designed to be accessible to participants with aphasia, was in large print and was supported with pictures.

**Table 1:** Participant feedback questions

1. How easy were the exercises without surface Electromyography?
2. How easy was it to understand the information on the screen?
3. How easy were the exercises with surface Electromyography?
4. Did surface Electromyography help you with the exercises?
5. How comfortable was surface Electromyography?
6. What was good about using surface Electromyography?
7. What was bad about using surface Electromyography?
8. Would you be happy to use surface Electromyography regularly?

### Data Analysis

Peak sEMG amplitudes were measured for each NS and ES task. The ES amplitudes were then normalised to the mean NS amplitude recorded within the same session, i.e. presented as a % of the mean normal swallow amplitude (%NS). Normalisation of sEMG data to a reference measurement taken from the same muscle in the same recording session is recommended to control for intrinsic and extrinsic factors affecting the raw signal that are unrelated to the level of muscle activation, for example the amount of fat and skin impedance and the orientation of the muscle fibres in relation to the recording electrodes [45]. This approach also presented how the NS and ES compared.

Normalised sEMG data and questionnaire responses are presented with means (SD) or medians (IQR) depending on distribution and type. Normalised sEMG data that was not normally distributed (Kolmogorov–Smirnov test, confirmed by histograms) was log-transformed (ln) and then normality was reassessed. This data was then used for all subsequent statistical analyses.

Ability to modify the trace for the ES compared with the normal swallow and the effects of biofeedback, session and participant group on ES performance were examined with two-way repeated measures analysis of variance (RM ANOVA) with the within subject factors “task” (i.e. normal swallow, ES with feedback (FB) and ES without FB) and “session” and the between subject factor “group” (i.e. healthy control *vs* stroke). Violations in sphericity were corrected with the Greenhouse–Geisser estimates of sphericity and post hoc pairwise comparisons were adjusted for multiple testing with the Bonferroni correction. The relationship between age and ability to benefit from biofeedback in healthy participants was examined with linear regression, with normalised FB ES amplitude as a percentage of ES without FB amplitude plotted against age.

For questionnaire data, within-group differences between questions were examined with the Wilcoxon Signed Rank Test. Differences between groups on questions were examined with the Kruskal–Wallis and the Mann–Whitney Test with adjustment for multiple testing with the Bonferroni correction.

## Results

### Participants

Fifteen acute stroke participants were recruited. Technical difficulties arose during the sEMG recording for one who was transferred to a different hospital before a second session could be conducted. Two further participants declined to have FEES. Therefore the results are based on 14 stroke participants, with baseline PAS scores available for 12 (Table 2). One stroke participant who was randomised to receive feedback first was too fatigued to complete the session protocol and therefore did not repeat the tasks without feedback. Seventeen healthy participants were recruited as the control group (Table 2). There were no significant differences between the two groups for age, sex or number of days between sessions (*p* > 0.05). The stroke group had significantly lower FOIS and Barthel scores (*p* < 0.001, Table 2). Due to the severity of the stroke participants’ dysphagia, only three could tolerate water; 5 participants had moistened oral care sponges, 4 had teaspoons of syrup thickened fluids, and two had teaspoons of yoghurt.

**Table 2:** Participant demographics

Group (*n*)	Stroke (14)	Healthy controls (17)	All healthy (85)
Age (years)	74.5 (61.3–83.3)	76.00 (74.5–81.5)	49.00 (29.0–70.0)
Sex			
Male	9	10	42
Female	5	7	43
Barthel	4.0 *(0.0–10.8)	20.0 (20.0–20.0)	20.00 (20.0–20.0)
Stroke type	R MCA infarct (5) L MCA infarct (3) L PICA infarct (1) L pontine infarct (1) R thalamic haemorrhage (1) R parietal haemorrhage (1) Multiple posterior circulatory infarcts (1) Multiple scattered lacunar infarcts (1)	n/a	n/a
Days from stroke to session 1	16.5 (7.0–1.3)	n/a	n/a
PAS on FEES	7.5 (5.3–8.0)	n/a	n/a
FOIS	4.0* (1.0–5.0)	7.0 (7.0–7.0)	7.00 (7.0–7.0)
Days between sessions	3.00 (1.0–4.0)	5.00 (1.5–7.0)	6.0 (4.0–7.0)

*PAS* penetration aspiration scale, *FOIS* functional oral intake

Medians and interquartile ranges are shown. Significant differences between the stroke group and both healthy groups are indicated by * (*p* ≤ 0.001) on Mann–Whitney *U* Test for independent samples

For the study of age-related changes, 85 healthy participants aged 18–89 years were recruited but 2 did not attend the second session (Table 2). The sEMG amplitudes for all participant groups were not normally distributed for either session or both combined (≤ 0.001), justifying log transformation for statistical analysis. Questionnaire data was collected from all who completed the second session: 83 healthy and 14 stroke participants.

#### Does Age Affect the Ability to Increase Submental Muscle Activity During the ES?

**Fig. 2:**
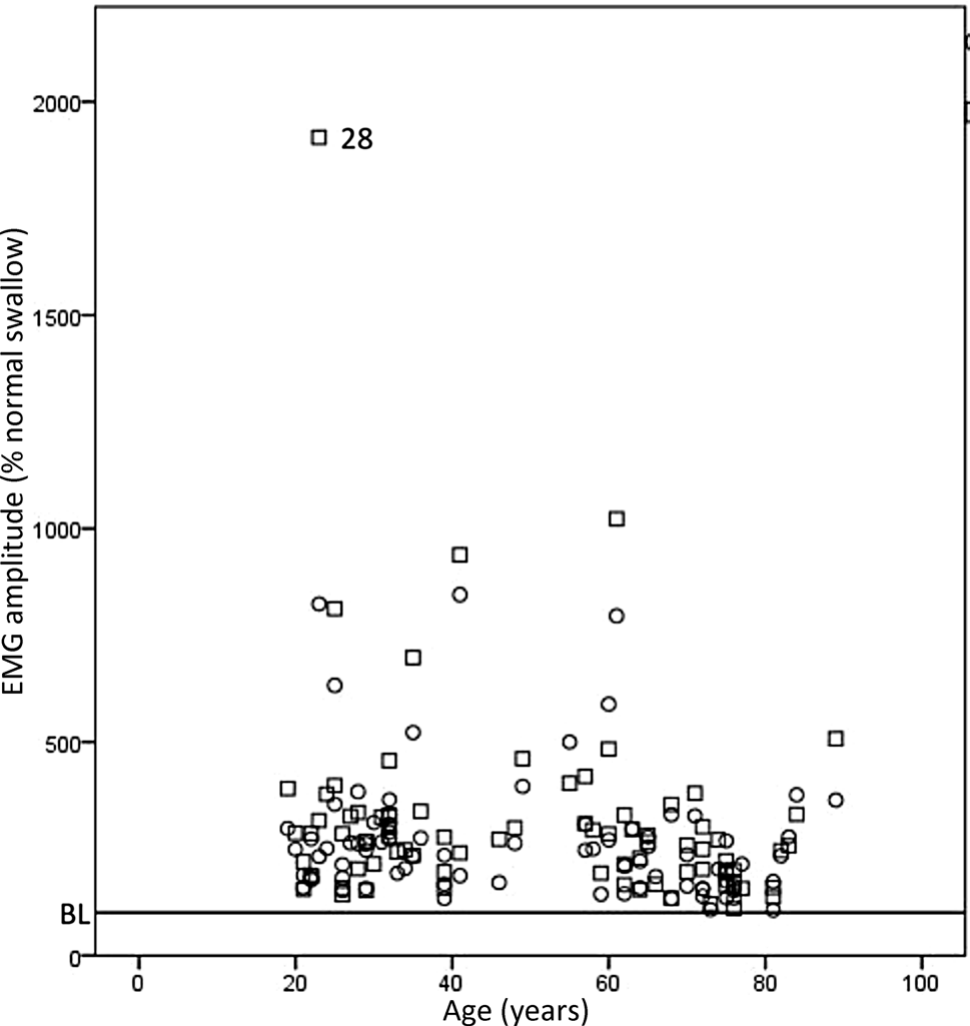
Age and mean normalised sEMG amplitude for the ES exercise in healthy participants across sessions 1&2. Open circle = ES without feedback, open square = ES with feedback. Line at 100% normal swallow represents baseline (BL), i.e. mean normalised normal swallow amplitude (*n* = 83). *ES* effortful swallow. Annotated data indicates outlier with participant’s identification code marked

There was no change in amplitude for the ES with or without feedback with age (*r* = 0.178, *p* = 0.110, Fig. 2). One participant produced markedly increased amplitude for ES compared with the others (participant 28). They also reported that the electrode and tape were “restrictive” on his swallow (see questionnaire data, following). It may be that the equipment was secured too tightly, which then led to electrode movement with hyo-laryngeal excursion and poor electrode contact contaminated the data. This participant’s data was removed from further analysis as it was a clear outlier.

#### Does Post-stroke Dysphagia Affect the Ability to Increase Submental Muscle Activity During the ES?

**Fig. 3:**
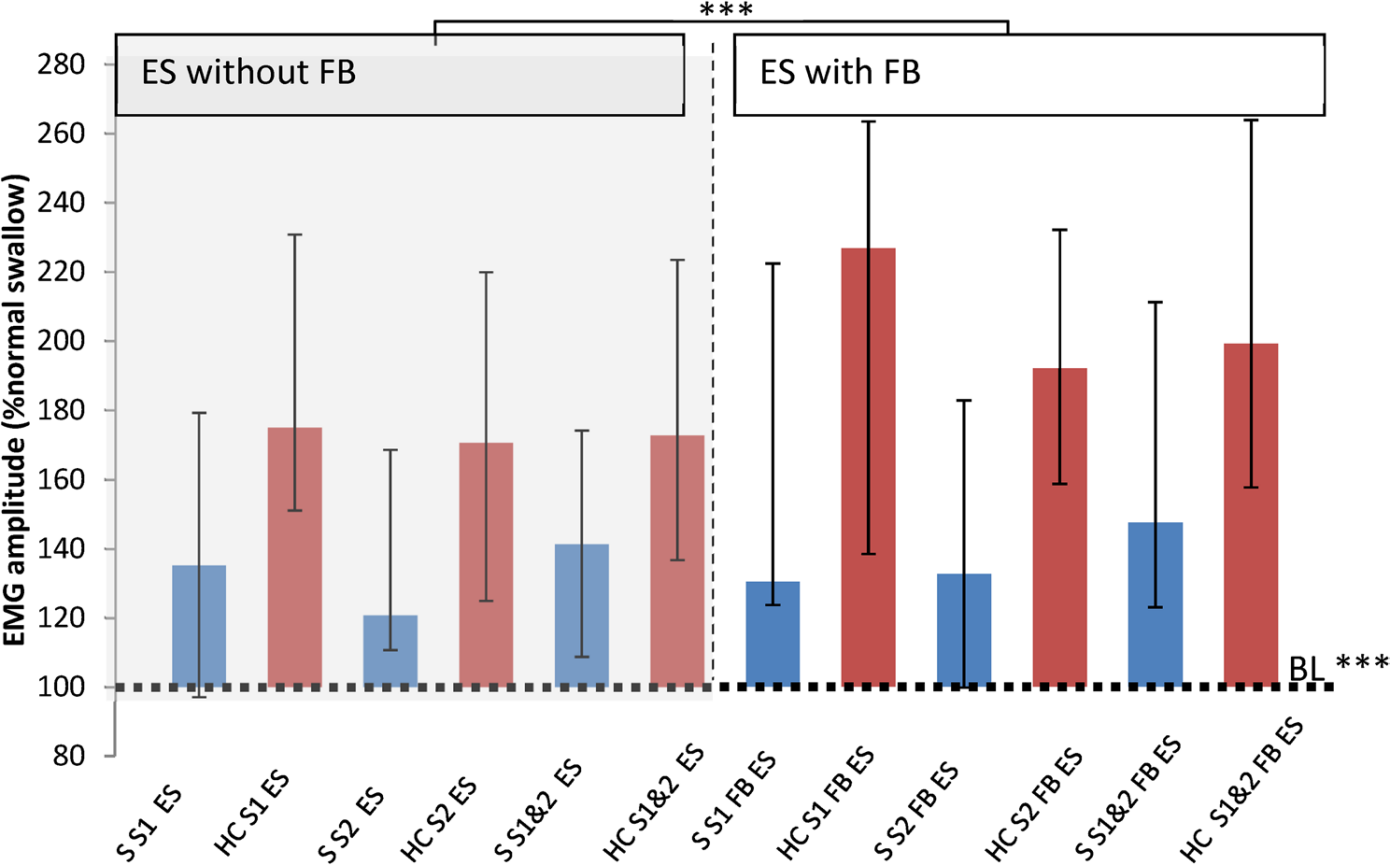
Effortful swallows (ES) with Feedback (FB) and without FB by stroke participants 'S' (n = 13) and healthy controls 'HC' (*n* = 17) for session 1 (S1) and 2 (S2) and for both combined (S1&2). Medians and IQR shown. Data is normalised to the normal swallow baseline (BL dotted line, i.e. 100%NS). There was a significant effect of task and FB with no effect of session. ***p < 0.001

**Fig. 4:**
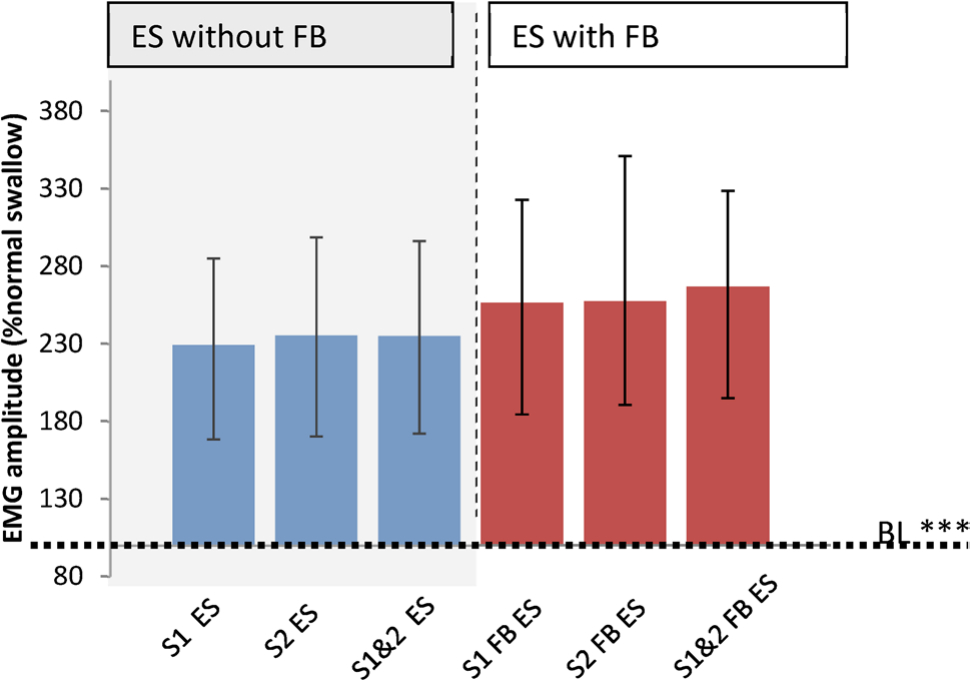
Healthy participants effortful swallow (ES) amplitude (*n* = 82). Medians and inter-quartile ranges shown. Dotted line at 100% NS and *BL* = mean normalised normal swallow baseline. Asterisks on *BL* = significant difference between ES and normal swallow. There was a significant effect of FB (***p < 0.001) and no effect of session

Healthy controls produced greater median normalised ES amplitudes than stroke participants in all conditions and sessions (Fig. 3); for example healthy controls performing ES with FB produced median amplitudes of 199.12% NS vs 147.53% NS by stroke participants across both sessions. However there was no significant main effect of participant group (*p* = 0.113). There was a significant main effect of swallowing task (ES vs NS); *F*(1.157, 32.385) = 43.202, p < 0.001. On post hoc tests, ES tasks resulted in significantly higher amplitudes than the normal swallow; for ES with FB the ln mean difference was 18.233 (SE 2.709, *p* < 0.001) and for ES without FB the ln mean difference was 13.964 (SE 2.064, *p* < 0.001). This indicates that both stroke participants and healthy controls were able to modify the sEMG trace above their normal level of swallowing activity for the ES exercise (Fig. 3, 4).

#### Does sEMG Biofeedback Improve the Performance of the ES by Healthy and Dysphagic Participants?

For all healthy participants (*n* = 82), there was again a significant main effect of swallowing task on amplitude (*F*(1.032,83.599) = 46.674, *p* < 0.001, Fig. 3) with no effect of session. Post hoc tests revealed that ES amplitudes were significantly larger with FB than without: ln mean difference 3.538 (SE 0.518, *p* < 0.001). The median for the ES with FB was 266.74% NS and for the ES without FB was 235.17% NS. For stroke participants and healthy age-matched controls there was no effect of participant group or session but ES amplitudes were significantly increased with FB; ln mean difference 4.324 (SE 1.042, *p* = 0.001, Fig. 3). Healthy controls median normalised amplitude for ES with FB was 199.12% NS vs 172.78% NS for ES without FB; for stroke participants the median normalised amplitudes were 147.53% NS vs 141.39% NS respectively. There was no difference with age on the effect of biofeedback on ES amplitude (*r*^2^ = 0.001, *p* = 0.93).

#### What do Participants Think About sEMG Biofeedback?

The majority of healthy (83%, *n* = 69) and stroke (86%, *n* = 12) participants reported that sEMG feedback helped them to complete the exercise and that they would be happy to use it regularly (99% *n* = 82 and 100% *n* = 14, respectively). Participants were asked what was good about sEMG feedback and frequent responses related to having visual feedback on performance and progress, having a target to aim for and it being interesting and enjoyable. No participants entered “nothing” in response to this question. The most frequent response to the question about what was bad about sEMG was “nothing”, by 47% (*n* = 39) of healthy and 79% (*n* = 11) of stroke participants. Other responses were that that the electrode placement felt odd and that the process was distracting (Table 3).

**Table 3:** Responses to the questionnaire about sEMG biofeedback by healthy (*n* = 83) and stroke (*n* = 14) participants

Group	What was good about using sEMG?	% (*n*)	What was bad about using sEMG?	% (*n*)
Healthy (*n* = 83)	Visual feedback about performance and progress/re-enforcing correct technique	38.6 (32)	Nothing	47.00 (39)
			Feels odd/unnatural/ “stiffening”/ felt like the restriction of the pad may have changed swallow	8.4 (7)
	Could see what I was trying to achieve and aim for/gave me a target/personal best	21.7 (18)		
			Distracting	6.0 (5)
	Interesting/fascinating	18.1 (15)	Abrasive skin preparation	3.6 (3)
	Made it fun/enjoyable	10.8 (9)	Taking off the electrodes	2.4 (2)
	Helped me to understand the exercise/swallowing	7.2 (6)	Confusing	2.4 (2)
			Fatigue	1.2 (1)
	Non invasive	4.8 (4)	Made me cough	1.2 (1)
	Quick and easy to set up	3.6 (3)	Used other muscles to complete task	1.2 (1)
	Helped motivate/encourage me	3.6 (3)	It is quite hard to swallow normally when you know you are being tested	1.2 (1)
	Easy to understand	3.6 (3)		
	Comfortable	3.6 (3)	Felt under pressure to meet target	1.2 (1)
	Being able to see the muscles working	2.4 (2)	Position of the screen above my head, would have been better at eye-level	1.2 (1)
	Screen clear/easy to see	2.4 (2)		
	No comment	6.0 (5)	Large equipment, small, portable version would be nicer	1.2 (1)
			Coordinating EMG, spoon and swallow together was hard at first	1.2 (1)
			No comment	4.8 (4)
Stroke (*n* = 14)	I could see how I was doing which was helpful	35.7 (5)	Nothing	78.6 (11)
	Made it a challenge/gives you a target	14.3 (2)	Didn't like smell of alcohol wipe	7.1 (1)
	Helps you know how to practise	7.1 (1)	Didn't like electrodes stuck	7.1 (1)
	You know what you have to do after the session	7.1 (1)	My feedback loop is not strong enough. Not clear what to do to improve things	7.1 (1)
	Very happy with the system	7.1 (1)		
	Motivating	7.1 (1)		
	Measurement of muscles	7.1 (1)		
	You (SLT) can see how I am doing	7.1 (1)		
	No comment	21.4 (3)		

**Fig. 5:**
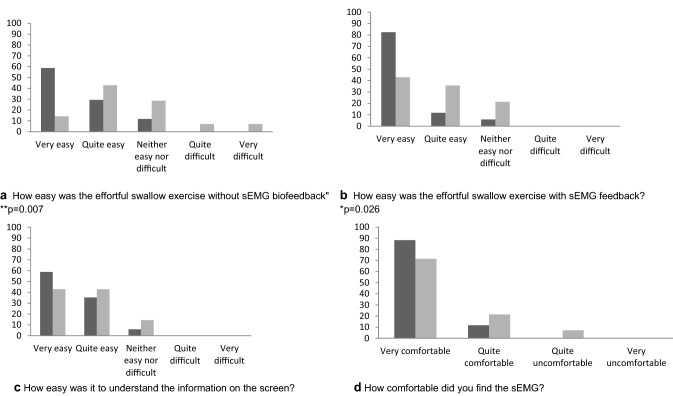
Responses to questions by stroke participants(pale grey bars, *n* = 14) and healthy controls (dark grey bars, *n* = 17). Significant differences in the spread of responses between participant groups are indicated *p ≤ 0.05 and **p ≤ 0.01

When stroke participants’ responses were compared with healthy age and sex matched controls (*n* = 15), the most frequent response to the question “How easy was the effortful swallow exercise without biofeedback?” was “very easy” for healthy controls (59%; *n* = 10) and “quite easy” for stroke participants (43%; *n* = 6), but responses were spread across the possible range for stroke participants, with 14% (*n* = 2) reporting that it was “quite” or “very difficult” (Fig. 5a). Stroke participants scored significantly higher for this question (i.e. reported they found the exercise more difficult than controls) (*z* = 2.69, *p* = 0.007). The responses to the question “How easy was the effortful swallow exercise with sEMG biofeedback” were more positive, with the most frequent response being “very easy” for both groups (82% of healthy and 43% of stroke participants) and no participants reporting it was difficult. Again, stroke participants responses indicated they found the exercise significantly more difficult than controls (*z* = 2.22, *p* = 0.026, Fig. 5b).

Both healthy and stroke participants reported finding the exercises significantly easier with the sEMG biofeedback than without (*z* = 2.24, *p* = 0.025 and *z* = 2.64, *p* = 0.008, respectively). There were no other statistically significant differences between the groups on the other questionnaire items and both groups indicated that they largely found the procedure comfortable and easy to understand (Fig. 5c, d). The majority (86%, *n* = 12) of stroke participants and all healthy controls reported that sEMG helped them with the exercise. All stroke participants and age and sex matched controls stated that they would be happy to use sEMG regularly if appropriate.

## Discussion

This study has shown for the first time that dysphagic stroke patients are able to significantly increase their muscle activity for the ES exercise. Incorporating sEMG biofeedback led to further significant increases in sEMG amplitudes and was considered a positive adjunct to therapy by participants. These findings are supportive of the potential application of the ES and sEMG biofeedback to treat swallowing impairments after stroke, justifying further work to examine therapy outcomes.

### Does Age Affect the Ability to Increase Submental Muscle Activity During the ES?

There was no change in the ability to modify sEMG activity for the ES with age. Conversely, Yeates et al. [31] found a non-significant reduction in ES amplitude with age but notably only one ES was elicited in their protocol and there was no practice/training period. In the present study, the mean of 6 trials was taken after a practice period. The lack of change with age was surprising considering the evidence of increased swallowing difficulties [46], pharyngeal wall atrophy [37] and reduced pharyngeal pressure generation [32, 47] with healthy ageing. This may reflect the non-specific nature of sEMG measurement, with the resultant signal representing the composite activity of a number of muscles, including the tongue [30], which may allow for compensation of individual muscle weakness. Consistent with the current findings, a study exploring tongue function found that normalising swallowing tongue pressures to the maximum tongue pressure negated previously reported age-related differences in functional reserve, and suggested that apparent deterioration in functional reserve is actually an artifact attributable to normal individual variation in tongue strength that is independent of age [48].

### Does Post-stroke Dysphagia Affect the Ability to Increase Submental Swallowing Activity During the ES?

All groups produced significantly greater amplitudes for the ES than NS, indicating that people with dysphagia from acute stroke, as well as healthy participants in a wide range of ages, can modify muscle activity during the ES. There was a clear trend for healthy controls to produce higher ES amplitudes, but this did not reach significance, which may be due to the small sample size and wide variability. Reduced ES amplitudes could be expected following stroke, reflecting muscle weakness and reduced voluntary activation. Promisingly, stroke participants’ ability to modify sEMG activity indicates they have some preserved functional reserve despite having relatively severe dysphagia (median PAS 7.5/8) and being in the acute stage of recovery. This provides support for the potential of ES as a task-specific exercise that challenges the system beyond normal levels of activity [7]. The normalised amplitudes achieved by the healthy participants could serve as targets for therapy programmes for dysphagic patients.

### Does sEMG Biofeedback Improve the Performance of the ES by Healthy and Dysphagic Participants?

All groups produced significantly greater sEMG amplitude for the ES with feedback. This provides strong support for using sEMG biofeedback as an adjunct in dysphagia therapy, further achieving the “overload” principle of rehabilitation [7]. Feedback is essential for motor learning [9] and yet informative and meaningful feedback is very difficult to deliver in dysphagia therapy as swallowing is a largely hidden activity. In assessing the ES, clinicians are otherwise restricted to subjective laryngeal palpation and feedback consists of subjectively describing how they feel the patient performed, which has questionable accuracy and meaning for the patient, especially considering the poor reliability of individual components of the clinical examination [12]. With sEMG feedback, both participants and clinicians are presented with objective, quantifiable data against which targets can be set and progress monitored which could facilitate both motivation and performance.

### What do Participants Think about sEMG Biofeedback?

The evaluation of patient experience has gained increased attention among healthcare providers, with an understanding that outcomes of care are improved if the experience is positive [49]. sEMG biofeedback has not previously been routinely offered in the UK or Ireland by SLTs working in stroke [29] and evaluating participants’ perceptions of the technique is indicated to establish acceptable and realistic treatment protocols.

The feedback from both stroke and healthy participants was very positive about sEMG. A large majority in both groups reported that it helped them to complete the ES and almost all reported that they would be happy to use it regularly, indicating that it is an acceptable and comfortable technique. A frequent response was that it gave them helpful visual feedback on performance and progress, reinforcing their technique, and that they benefitted from having a target to aim for. Biofeedback has been described as enabling participants to be more actively involved in therapy, providing them with objective evidence for motivation and thereby improving outcome [20]. The results of the present study support this theory from the perspectives of the participants themselves.

The largest proportion of respondents reported that there was nothing negative about the procedure but the comments that were obtained are helpful for improving treatment protocols. Although participant comfort was always checked at the beginning of the session, establishing ongoing feedback during the exercises would have enabled re-evaluation and reassurance about electrode placement to avoid the “odd” or “restrictive” sensation reported by a few. Clinicians should be mindful of the potential negative impact of sEMG biofeedback in diverting attention away from the sensation of the swallow itself as a small group of healthy participants reported that sEMG biofeedback was distracting or confusing. This highlights the need to evaluate the benefits/disadvantages of biofeedback on an individual basis.

### Strengths and Limitations

A limitation of this study was the small sample size of stroke participants; however as this was a Phase 1 study, the results suggest the potential of the ES and sEMG biofeedback in enhancing fundamental mechanisms of functional swallowing recovery, justifying further work. While all stroke participants had no history of previous stroke/neurological impairment, participants were severely impaired based on their Barthel scores [43] and the sample was varied in terms of type and location of stroke. However, they were intentionally representative of the clinical population and one purpose of this study was to collect data from a “typical” inpatient acute stroke population who would receive dysphagia therapy in a standard clinical setting.

Due to risks of aspiration in the stroke group, only three participants were able to swallow teaspoons of water and so the other participants were given other (safe) consistencies. Data collection commenced with an established local protocol with healthy participants prior to recruiting the first stroke participant and the degree of impairment of the stroke participants was not foreseen. Therefore the different groups were not given the same distribution of consistencies. However, the protocol was aimed to elicit the most natural swallowing possible, which justified participants having their safest consistency. Furthermore, normalising the data should have controlled for differences based on consistency.

A possible limitation of the questionnaire was that it was completed after just two short sessions of sEMG biofeedback and therefore participants were not given much experience of the technique. By specifically asking them to respond to the questions about what was good and bad about the technique, they were arguably forced to think of an answer when they might have responded differently with a more open question. However, this approach was felt to enrich the number of responses obtained. There was a risk of response bias with the participants potentially responding positively to please the researcher. However, they were told that the purpose of the study was to evaluate a new technique in which the researcher had no vested interest, to see if it was worth recommending to clinicians in the future. They were also reassured that questionnaire forms remained anonymous and were analysed together at a later date. Therefore the risk of response bias was minimised as far as possible. This study was not designed to enable full thematic analysis of participant feedback and this would be a useful part of any future studies examining patient experience and outcome from treatment.

This study was not designed to determine patient outcome in response to therapy with the ES and this requires further study. However, this current work addressed an important preliminary question of whether patients with significant dysphagia are able to adapt their muscle activity during the exercise and the impact of biofeedback on this, therefore supporting further work on therapy outcome.

A strength of this study was including physiological information about the effects of the ES exercise with feedback from participants. Clinicians cite low motivation as the most common reason that they feel patients do not improve as a result of dysphagia therapy [29]. This study therefore sought to determine whether the ES with sEMG feedback was acceptable to participants. The positive feedback from participants, together with the encouraging physiological effects, strengthen the evidence for this technique.

## Conclusion

For the first time the ability of dysphagic stroke participants to modify muscle activity during the ES has been shown. This justifies further study of the ES as it complies with the “overload” and “use it or lose it” principles of rehabilitation [7, 50]. Furthermore, both healthy and stroke participants produced more muscle activity with feedback, indicating that it could be a valuable adjunct to ES training. The results of the participant questionnaire are encouraging with respect to the acceptability of sEMG biofeedback to patients. Participants were positive in their comments and expressed the perceived benefits of the technique in terms of feedback, monitoring and target setting. These findings support consideration of sEMG biofeedback as a useful adjunct in dysphagia therapy, which may both improve patient enjoyment and motivation as well as enhancing performance. Further trials are now indicated to determine the functional benefit of these treatments.
